# A New Genetic Diagnostic for Enlarged Vestibular Aqueduct Based on Next-Generation Sequencing

**DOI:** 10.1371/journal.pone.0168508

**Published:** 2016-12-20

**Authors:** Yalan Liu, Lili Wang, Yong Feng, Chufeng He, Deyuan Liu, Xinzhang Cai, Lu Jiang, Hongsheng Chen, Chang Liu, Hong Wu, Lingyun Mei

**Affiliations:** 1 Department of Otolaryngology, Xiangya Hospital, Central South University, Changsha, Hunan, China; 2 Province Key Laboratory of Otolaryngology Critical Diseases, Xiangya Hospital, Central South University, Changsha, Hunan, China; 3 Department of Otolaryngology, The Second Affiliated Hospital of Zhengzhou University, Zhengzhou, Henan, China; 4 The State Key Laboratory of Medical Genetics, Central South University, Changsha, Hunan, China; 5 R&D Department, Genesky Diagnostics Inc., Suzhou, Jiangsu, China; NIDCR/NIH, UNITED STATES

## Abstract

Enlarged vestibular aqueduct (EVA) is one of the most common congenital inner ear malformations and accounts for 1–12% of sensorineural deafness in children and adolescents. Multiple genetic defects contribute to EVA; therefore, early molecular diagnosis is critical for EVA patients to ensure that the most effective treatment strategies are employed. This study explored a new genetic diagnosis method for EVA and applied it to clinic diagnoses of EVA patients. Using next-generation sequencing technology, we set up a multiple polymerase chain reaction enrichment system for target regions of EVA pathogenic genes (SLC26A4, FOXI1, and KCNJ10). Forty-six EVA samples were sequenced by this system. Variants were detected in 87.0% (40/46) of cases, including three novel variants (SLC26A4 c.923_929del, c.1002-8C>G, and FOXI1 c.519C>A). Biallelic potential pathogenic variants were detected in 27/46 patient samples, leading to a purported diagnostic rate of 59%. All results were verified by Sanger sequencing. Our target region capture system was validated to amplify and measure SLC26A4, FOXI1, and KCNJ10 in one reaction system. The result supplemented the mutation spectrum of EVA. Thus, this strategy is an economic, rapid, accurate, and reliable method with many useful applications in the clinical diagnosis of EVA patients.

## Introduction

Enlarged vestibular aqueduct (EVA; MIM 600791) is an autosomal recessive genetic disease causing congenital inner ear malformation that accounts for 1–12% of sensorineural deafness in children and adolescents [1]. EVA can be divided into syndromic EVA (mostly Pendred syndrome [PDS]; MIM274600) and the more common nonsyndromic EVA (DFNB4; MIM 600791) depending on the presence of other inner ear malformations or diseases.

The SLC26A4 (DFNB4; MIM 605646) gene encodes pendrin, which is expressed in the inner ear and is responsible for EVA symptoms. In many EVA patients, SLC26A4 screening identified two disease-causing allele variants[2]. More than 300 variants have been identified in the SLC26A4 gene with EVA or PDS (www.healthcare.uiowa.edu/labs/pendredandbor), and each ethnic population has a different and diverse variant spectrum with their own specific mutation hot spots [3,4]. The function of SLC26A4 also has been explored in Pendrin knockout mice that recapitulate the pathology observed in humans: profound deafness and bulged endolymphatic spaces of the inner ear with striking [5]. Recent studies found that the SLC26A4 promoter contains a key transcriptional regulatory element that binds FOXI1 (MIM 601093), a transcriptional activator of the gene [6]. Additionally, double heterozygosity of SLC26A4 and KCNJ10 (MIM 602208) was identified in individuals with an EVA phenotype from two families, linking KCNJ10 variants with EVA [7].

Routine clinical examinations to diagnose EVA involve audiological tests (*e*.*g*., pure tone audiometry, acoustic immittance, auditory steady-state response, and auditory brainstem response) and temporal bone imaging (*e*.*g*., computed tomography and magnetic resonance imaging) to reveal expansile vestibular or endolymphatic sac. EVA manifests clinically as fluctuating or progressive sensorineural hearing loss, ranging from mild to profound deafness [8], so most patients are clinically diagnosed when their hearing is already poor. Therefore, early clinical genetic diagnoses of EVA patients are critical to clarify the molecular etiology and implement the appropriate disease control and prevention responses, such as avoiding head trauma, getting cold and noise stimulation. When patients with hearing loss were diagnosed of EVA, they can choose hearing aid or artificial cochlear implantation as soon as possible.

EVA molecular diagnoses have traditionally relied upon Sanger sequencing. More recently, variant detection systems using denaturing high-performance liquid chromatography (DHPLC) have been developed for PDS screening [9,10]. Array-based variant screenings also are a rapid and efficient technique to detect known variants. A recently developed genome-wide association approach to detect loci affecting PDS susceptibility used 597 genotyped sows with 62,163 single nucleotide polymorphisms (SNPs) [11]. We developed a microarray to detect 240 variants underlying syndromic and nonsyndromic sensorineural hearing loss, including 11 distinct variants in SLC26A4 [12]. However, all of these EVA genetic diagnosis strategies rely upon either full gene sequencing or test for common variants in only the SLC26A4 gene. These approaches are not optimal, as they do not allow for all EVA-associated genes to be simultaneously examined. Furthermore, these techniques are both time-consuming and costly, limiting their clinical application. Recent developments in next-generation sequencing (NGS) have widely expanded its use in scientific research and clinical fields, promoting the development of assays to rapidly and cost-effectively sequence all genes and noncoding regions of interest. NGS can provide more precise information about genetic causes of disease and recurrence risks, ultimately leading to better treatment [13,14]. Several NGS methods have been developed and clinically applied for hereditary hearing loss [14,15,16].

We developed a molecular diagnosis for EVA based on multiple polymerase chain reaction (PCR) targeted enrichment and NGS that includes three known EVA-associated genes. This genetic diagnostic can be extended to clinical practices and has the power to advance simple genetic screening into genetic diagnoses.

## Materials and Methods

### Subjects

A cohort of 46 sporadic Chinese probands diagnosed with EVA was recruited between 2014 and 2016 from the Otolaryngology Department of Xiangya Hospital, Central South University, including 32 males and 14 females, the average age was 6, range from 1 to 26 years old (Table 1). A detailed medical history was available for each proband. Every participant was examined thoroughly, including systemic and specialized physical examination, electric otoscopy and audiological assessment (pure tone audiometry, acoustic immittance, auditory steady-state response and auditory brainstem response). High Resolution Computed Tomography (HRCT) scanning of temporal bone and Magnetic Resonance Hydrography (MRH) examination of inner ear were performed on all the patients. Inclusion criteria of EVA patients were HRCT shows significant bone defect on posterior border of petrosum. The width of external opening of vestibular aqueduct is more than 1.5 millimeter, or the width of middle opening is over 2 millimeter. MRH shows expanded endolymphatic sac. Syndromic features were not detected. The controls consisted of 100 unrelated healthy Chinese volunteers with normal hearing and without another genetic disease. All patients and controls were ethnically Chinese. Written informed consent was obtained from all the participants or their parents (when participants were under 18 years old).and the research was approved by the Ethic Committee of the Xiangya Hospital of Central South University and is compliant with the Code of Ethics of the World Medical Association[17].

**Table 1:** Details of Phenotype of the 46 patients.

Patient number	Gender	Age^a^	Pre/Postlingual	Degree of hearing loss^b^	Evolution of hearing loss	IEM
01	Male	6	Prelingual	Profound	Progressive	EVA
02	Male	7	Prelingual	Profound	Fluctuating	EVA
03	Male	2	Prelingual	Profound	Stable	EVA
04	Male	13	Prelingual	Severe	Fluctuating	EVA
05	Female	10	Prelingual	Severe	Progressive	EVA
06	Female	5	Prelingual	Profound	Fluctuating	EVA
07	Female	6	Prelingual	Severe	Stable	EVA
08	Female	11	Postlingual	Profound	Fluctuating	EVA
09	Female	2	Prelingual	Profound	Stable	EVA
10	Female	5	Prelingual	Profound	Progressive	EVA
11	Female	1	Prelingual	Severe	Stable	EVA
12	Female	15	Postlingual	Severe	Progressive	EVA
13	Female	1	Prelingual	Profound	Stable	EVA
14	Female	2	Prelingual	Profound	Stable	EVA
15	Female	2	Prelingual	Profound	Stable	EVA
16	Female	6	Postlingual	Profound	Progressive	EVA
17	Female	13	Prelingual	Profound	Fluctuating	EVA
18	Female	7	Postlingual	Severe	Fluctuating	EVA
19	Female	6	Prelingual	Profound	Progressive	EVA
20	Female	3	Prelingual	Profound	Stable	EVA
21	Female	2	Prelingual	Severe	Stable	EVA
22	Female	4	Prelingual	Profound	Fluctuating	EVA
23	Female	2	Prelingual	Profound	Stable	EVA
24	Female	1	Prelingual	Profound	Stable	EVA
25	Female	6	Prelingual	Profound	Fluctuating	EVA
26	Female	6	Prelingual	Severe	Progressive	EVA
27	Female	4	Prelingual	Profound	Fluctuating	EVA
28	Female	4	Prelingual	Profound	Stable	EVA
29	Female	4	Prelingual	Profound	Stable	EVA
30	Female	6	Prelingual	Severe	Progressive	EVA
31	Female	10	Prelingual	Profound	Progressive	EVA
32	Female	11	Prelingual	Severe	Fluctuating	EVA
33	Female	1	Prelingual	Profound	Stable	EVA
34	Female	3	Prelingual	Profound	Stable	EVA
35	male	13	Prelingual	Profound	Progressive	EVA
36	male	4	Prelingual	Profound	Stable	EVA
37	Female	15	Prelingual	Severe	Fluctuating	EVA
38	male	10	Prelingual	Profound	Fluctuating	EVA
39	Female	12	Prelingual	Profound	Progressive	EVA
40	male	2	Prelingual	Profound	Stable	EVA
41	male	26	Postlingual	Severe	Fluctuating	EVA
42	Female	4	Prelingual	Profound	Stable	EVA
43	male	5	Prelingual	Profound	Stable	EVA
44	Female	6	Prelingual	Severe	Fluctuating	EVA
45	male	6	Prelingual	Profound	Progressive	EVA
46	male	5	Prelingual	Severe	Stable	EVA

^a^ Age (in years) at genetic consultation for study inclusion.

^b^ Profound means hearing threshold consultation foeans hearing threshold 71-90dB, Moderate means hearing threshold 41-55dB, Mild means hearing threshold 26-40dB.

IEM: inner ear malformation

### Design of Captured Target Genome Regions and Multiple PCR Enrichment System

**Fig 1:**
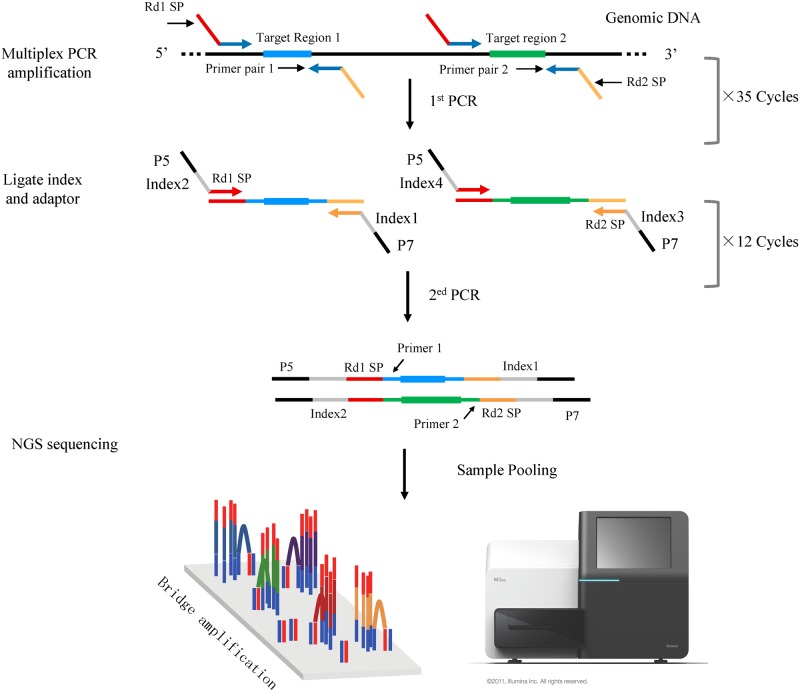
Multiple PCR Target Enrichment and Next-Generation Sequencing.

Genomic DNA was extracted from peripheral blood using standard phenol-chloroform protocols and stored at -20°C. After purification and quality testing, multiple PCR enrichment was performed in accordance with the special reaction conditions developed in this study. The target genome regions of the three candidate genes (SLC26A4, FOXI1, and KCNJ10) were designed to include their promoter regions (~500 bp), 5`untranslated region (5`UTR), coding regions, splice sites (~8 bp), and 3' untranslated region (3' UTR) (Table 2). Thirty-nine primer pairs were designed using FastTarget Primer (V5.0.1) software developmented by Genesky, with the most stringent conditions (no SNPs in primer annealing region, amplicon length between 230–315 bp, GC content between 30 and 80%). These primers were synthesized and assigned into four multiplex PCR panels to amplify all the target regions of the three genes. The first round enrichment amplification reactions were carried out on a ABI 2720 Thermal Cycler (Life Technologies Corporation, USA) with following cycling program: 95°C for 2 min; 11 cycles of 94°C for 20 s, 63°C-0.5°C per cycle for 40 s, 72°C for 1mins; 24 cycles of 94°C for 20 s, 65°C for 30 s, 72°C for 1 mins; 72°C for 2 min. In the second round, four multiple PCR reaction products from the first round were mixed, and a pair of universal primer with an added index sequence was used to amplify for distinguishing different samples. The EVA sequencing library was constructed after the two rounds of amplification (Fig 1).

**Table 2:** EVA-associated genes and their PCR target regions.

Gene Name	NM_Accession	Exon Number	Gene Length (bp)	mRNA Length (bp)	Target regions length
FOXI1	NM_012188.4	2	3813	2296	619|coding+5'UTR,1677(563)|coding+3'UR
KCNJ10	NM_002241.4	2	32795	5306	5066(1141)|coding+3'UTR,240|5'UTR
SLC26A4	NM_000441.1	21	57175	4930	221|5'UTR,167|coding+5'UTR,140,111,185,165,153,83,148,114,78,96,107,70,93,96,231,55,146,84,2387(24)|coding+3'UTR

### Next-Generation Sequencing

The PCR production of each sample was labeled with 8bp index, all the libraries of each sample were pooled. After Cluster Generation and hybridization of sequencing primer, base incorporation was carried out on MiSeq Benchtop Sequencer (Illumina, Inc, San Diego, CA) in one single lane following the manufacturer's standard cluster generation and sequencing protocols, for 608 cycles of sequencing per read to generate paired-end reads including 300bp at each end and 8 bp of the index tag. The average effective sequencing depth of every sample was300× and sequencing depth of all bases was above 20×.

### Variants Analysis

Sequencing reads were aligned to hg19 using the Burrows–Wheeler Aligner (BWA) [18]. SNV calling was performed using both GATK and Varscan programs [19, 20], and the called SNV data were then combined. The Annovar program was used for SNV annotation [21]. The functional effect of non-synonymous SNVs was assessed by the PolyPhen-2, SIFT, and MutationTaster[22,23,24]. Non-synonymous SNVs with SIFT score of <0.05, Polyphen-2 score of >0.85 or MutationTaster score of >0.85 were considered as significant of not being benign. To sort potentially deleterious variants from benign polymorphisms, perl scripts were used to filter the SNVs against those of dbSNP135. Any SNV recorded in dbSNP135 and with a minor allele frequency of ≥1% in Chinese from 1000 genome database was considered as benign polymorphisms and therefore removed for subsequent analysis. We also test all the variants for their allele frequency in the Exac exome variant database (http://exac.broadinstitute.org/) to further support the novel variants being pathogenic.

### Sanger Sequencing

Variants selected and suspected to be pathogenic were confirmed by Sanger sequencing. Parental samples were used for segregation analysis of the sequence variants identified in the index proband via Sanger sequencing. In addition, 100 controls were sequenced for the variants detected to evaluate the population-wide incidence of the novel variants. Data were analyzed using DNASTAR software program (DNASTAR, Inc., Madison, Wisconsin, US).

## Results

All probands were diagnosed as DFNB4. Possibly pathogenic gene variants were found in 40 of 46 cases (87%). Thirty-eight cases carried SLC26A4 variants and two cases carried FOXI1 variants. KCNJ10 gene variants were not detected. By analyzing variant results of all the available DNA of patients’ parents, we found that 27 cases conformed to cosegregation principles, including 19 compound heterozygous, two homozygous variants and six heterozygous (Table 3), leading to a purported diagnostic rate of 59%.

**Table 3:** SNVs detected in 46 EVA probands.

Patient number	SLC26A4	FOXI1	KCNJ10	Genotype	Inheritance status^a^	
					Mat	Pat
01	c.919-2A>G	wt	wt	HE	N/A	N/A
02	wt	c.519C>A	wt	HE	wt	wt
03	c.1343C>T;c.2168A>G	wt	wt	Comp HE	c.1343C>T	c.2168A>G
04	c.919-2A>G;c.1975G>C	wt	wt	Doub HE	N/A	N/A
05	c.2T>C;c.269C>T	wt	wt	Doub HE	wt	c.269C>T
06	c.1229C>T	wt	wt	HOM	N/A	N/A
07	wt	wt	wt	-	N/A	N/A
08	c.754T>C;c.919-2A>G	wt	wt	Comp HE	c.754T>C	c.919-2A>G
09	c.919-2A>G;c.1229C>T	wt	wt	Comp HE	c.919-2A>G	c.1229C>T
10	wt	wt	wt	-	N/A	N/A
11	wt	c.716C>T	wt	HE	wt	c.716C>T
12	c.2168A>G	wt	wt	HE	wt	c.2168A>G
13	c.919-2A>G	wt	wt	HE	c.919-2A>G	wt
14	c.919-2A>G	wt	wt	HE	c.919-2A>G	wt
15	c.109G>T;c.1079C>T	wt	wt	Doub HE	wt	c.109G>T
16	c.919-2A>G	wt	wt	HOM	c.919-2A>G	c.919-2A>G
17	c.919-2A>G	wt	wt	HOM	c.919-2A>G	c.919-2A>G
18	c.919-2A>G;c.2000T>C	wt	wt	Comp HE	c.2000T>C	c.919-2A>G
19	c.919-2A>G;c.2000T>C	wt	wt	Comp HE	c.2000T>C	c.919-2A>G
20	c.754T>C;c.919-2A>G	wt	wt	Comp HE	c.754T>C	c.919-2A>G
21	c.1174A>T;c.1716T>A	wt	wt	Doub HE	N/A	N/A
22	c.422T>C;c.1229C>T	wt	wt	Comp HE	c.1229C>T	c.422T>C
23	c.1174A>T;c.1229C>T	wt	wt	Comp HE	c.1174A>T	c.1229C>T
24	wt	wt	wt	-	N/A	N/A
25	wt	wt	wt	-	N/A	N/A
26	c.589G>A;c.919-2A>G	wt	wt	Comp HE	c.589G>A	c.919-2A>G
27	c.919-2A>G;c.1547dupC	wt	wt	Comp HE	c.1547dupC	c.919-2A>G
28	c.919-2A>G;c.1547dupC	wt	wt	Comp HE	c.1547dupC	c.919-2A>G
29	c.1173C>A;c.1229C>T	wt	wt	Doub HE	N/A	N/A
30	c.919-2A>G;c.1786C>T	wt	wt	Comp HE	c.919-2A>G	c.1786C>T
31	c.754T>C;c.919-2A>G	wt	wt	Comp HE	c.754T>C	c.919-2A>G
32	c.754T>C	wt	wt	HE	c.754T>C	c.919-2A>G
33	wt	wt	wt	-	N/A	N/A
34	c.2162C>T	wt	wt	HE	wt	c.2162C>T
35	c.919-2A>G; c.923_929del	wt	wt	Comp HE	c.919-2A>G	c.923_929del
36	c.754T>C;c.1229C>T	wt	wt	Comp HE	c.754T>C	c.1229C>T
37	c.1229C>T;c.2168A>G	wt	wt	Comp HE	c.1229C>T	c.2168A>G
38	c.1229C>T;c.2168A>G	wt	wt	Comp HE	c.1229C>T	c.2168A>G
39	c.919-2A>G;c.1694dupA	wt	wt	Doub HE	N/A	N/A
40	c.919-2A>G	wt	wt	HE	N/A	N/A
41	c.919-2A>G	wt	wt	HE	N/A	N/A
42	c.919-2A>G;c.2168A>G	wt	wt	Comp HE	c.919-2A>G	c.2168A>G
43	c.919-2A>G	wt	wt	HE	N/A	N/A
44	wt	wt	wt	-	N/A	N/A
45	c.919-2A>G;c.1002-8C>G	wt	wt	Doub HE	N/A	N/A
46	c.919-2A>G;c.1229C>T	wt	wt	Comp HE	c.1229C>T	c.919-2A>G

^a^: Whether the variant was inherited from the parents or not;

Mat:maternal; Pat:paternal; HE: heterozygous; HOM: homozygous; Comp HE: compound heterozygous; Doub HE: double heterozygote, which need further analyses to conform that the two heterozygous mutations are located in one allele or two alleles respectively. wt: wild-type; N/A: data was not available.

We identified a total of 24 potentially pathogenic variants in these three genes (Table 4), including three novel variants (SLC26A4 c.923_929del, c.1002-8C>G and FOXI1 c.519C>A), which were absent in 100 control subjects and not reported in the dbSNP, 1000 Genomes Project database and the Exac exome variant database. To see if these mutations were *de novo*, we also sequenced their parental DNA, and found that the variant FOXI1 c.519C>A was not inherited from the parents. All the three novel variants were uploaded to the Leiden Open Variation Database (http://www.lovd.nl/3.0/home).

**Table 4:** Summary of variants.

Gene	Sample Number	Exons/ Introns	Variants Type	Variants	Amino Acid Change	References
FOXI1	1	exon1	Missense	c.519C>A	p.H173Q	*de novo*
FOXI1	1	exon2	Missense	c.716C>T	p.P239L	[25]
SLC26A4	1	exon2	Missense	c.2T>C	p.M1T	[26]
SLC26A4	1	exon2	Missense	c.109G>T	p.E37X	[27]
SLC26A4	1	exon3	Missense	c.269C>T	p.S90L	[3]
SLC26A4	1	exon5	Missense	c.422T>C	p.F141S	[28]
SLC26A4	1	exon5	Missense	c.589G>A:	p.G197R	[27]
SLC26A4	5	exon6	Missense	c.754T>C	p.S252P	[3]
SLC26A4	24	IVS7	Splicing	c.919-2A>G	-	[29]
SLC26A4	1	exon8	Deletion	c.923_929del	p.308_310del	Novel
SLC26A4	1	IVS8	Splicing	c.1002-8C>G	-	Novel
SLC26A4	1	exon9	Missense	c.1079C>T	p.A360V	[30]
SLC26A4	1	exon10	Missense	c.1173C>A:	p.S391R	[31]
SLC26A4	2	exon10	Missense	c.1174A>T	p.N392Y	[3]
SLC26A4	9	exon10	Missense	c.1229C>T	p.T410M	[32]
SLC26A4	1	exon12	Missense	c.1343C>T	p.S448L	[33]
SLC26A4	2	exon14	Insertion	c.1547dupC	p.F515fs	[3]
SLC26A4	1	exon15	Insertion	c.1694dupA	p.F565fs	[34,35,36]
SLC26A4	1	exon16	Missense	c.1716T>A	p.F572L	[37]
SLC26A4	1	exon16	Missense	c.1786C>T	p.Q596X	[38]
SLC26A4	1	exon17	Missense	c.1975G>C	p.V659L	[39]
SLC26A4	2	exon17	Missense	c.2000T>C	p.F667S	[40]
SLC26A4	1	exon19	Missense	c.2162C>T	p.T721M	[41]
SLC26A4	5	exon19	Missense	c.2168A>G	p.H723R	[4]

Novel refers to variants were absent in 100 control subjects and not reported in the dbSNP, 1000 Genomes Project database and the Exac exome variant database.

*de novo* refers to variants were absent in the parents, 100 control subjects and not reported in the dbSNP, 1000 Genomes Project database and the Exac exome variant database.

Of these variants, 22 were SLC26A4 variants and two were FOXI1 variants. Twenty-four variants included 19 missense, two insertions, one deletion, and two splicing variants. In the SLC26A4 gene, 19 compound heterozygous variants (50%), nine heterozygous variants (23.7%), and three homozygous variants (7.9%) were detected. Besides, we detected seven double heterozygote (18.4%), including five patients whose parents’ DNA was not available and two patients(05 and 15) carried two heterozygote, one of which derived from the paternal transmission, however, the other heterozygote was not inherited from the parents. We cannot be sure that the two heterozygous mutations of the seven patients are located in one allele or two alleles respectively, which needs further analyses. Both FOXI1 gene variants were heterozygous.

Sanger sequencing completely verified the NGS results, indicating that the NGS accuracy rate was 100% in our study.

## Discussion

EVA is an autosomal recessive hereditary disease with obvious genetic heterogeneity that complicates investigations into its molecular mechanism. Currently studies suggest that an SLC26A4 biallelic variant (compound heterozygous or homozygous) was the main cause of EVA and PDS. EVA patients carrying SLC26A4 biallelic variants usually can be verified by videography diagnosis [27]. In our research, 50% of cases had compound heterozygous variants, 23.7% had heterozygous variants, and 7.9% had homozygous variants, consistent with previous studies. EVA patients carrying SLC26A4 monoallelic variants might only be carriers. However, there were a considerable number of EVA patients who carried SLC26A4 monoallelic variants or variants not detected in SLC26A4. Some arguments support that probably there are other undetected mutations harboring in the promoter region or in a potential splice site of intron of the second SLC26A4 allele, which was not searched in the present study, or there might be a digenic pattern of inheritance with the implication of a second gene [6]. In addition, some researchers suggested that the interaction between genetic and environmental factors may play a role in the pathogenic process of EVA [42].

To date, three genes have been associated with EVA: SLC26A4, FOXI1, and KCNJ10. Variants in SLC26A4 reportedly account for about 50% of PDS and nonsyndromic EVA cases [43], while FOXI1 and KCNJ10 account for only <1% of all cases [44]. Our result indicates that 82% of patients had SLC26A4 variants, further confirming that SLC26A4 is the most common pathogenic gene of EVA. At the time of writing, more than three hundred SLC26A4 variants have been reported (http://www.hgmd.cf.ac.uk/ac/index.php). In this study, we identified two novel mutations of SLC26A4 were not reported in the NCBI dbSNP, 1000 Genomes Project database and the Exac exome variant database, of which, a deletion (c.923_929del TAATTGC) was predicted to cause frameshift and produce truncated proteins by premature stops. The truncated region caused by the variation was located in a highly conserved region among mammals and located in the predicted SulP(high affinity sulphate transporter 1) domain, which were predicted to be disease causing by MutationTaster. In addition, a splice site change (c.1002-8C>G) of SLC26A4 was reported for the first time in this study. The variation was predicted to cause aberrant splicing and considered as pathogenic by MutationTaster. It was confirmed that SLC26A4 c.1002–4 C>G was contributed to PDS by mRNA studies revealing the splice mutation resulted in a putative truncated protein [45]. Interestingly, the novel variation (c.1002-8C>G) discovered in our study adjacent to the reported mutation (c.1002–4 C>G), which is possible impairs the same functional region of SLC26A4 lead to EVA by causing a frameshift and introduction of a premature stop codon. Functional analyses are suggested to be completed.

Though FOXI1 and KCNJ10 have been confirmed to be related to EVA, most research devoted to these genes has failed to find specific pathogenic variants through large sample screening studies [46,47]. Two FOXI1 gene variants were detected in our work and one KCNJ10 variant (c.812G>A) was detected in another study [48]. FOXI1 can activate and regulate the transcription of SLC26A4 gene by combined with two binding sites, FBS1 and FBS2, in promoter region of SLC26A4. A missense mutation (c.519C>A) of FOXI1 was reported for the first time in this study, which was absent in the parents, 100 control subjects and not reported in the dbSNP database. This variant lies within the conserved forkhead DNA-binding domain. The significance of variant located in this domain has been substantiated that compromise FOXI1 transactivation ability of SLC26A4 expression and are causally related to disease phenotype in EVA patients [6]. Therefore, it is possible that the mutation discovered in our study impairs its ability to activate SLC26A4 transcription. Additionally, the missense variant was predicted as disease causing by MutationTaster, a potential detrimental effect at the EVA phenotype is still possible to hypothesize. Functional study will be performed for verification in the future.

Several methods have been traditionally used for deafness gene detection (*e*.*g*., Sanger sequencing, restriction enzyme fingerprinting-single strand conformation polymorphism analysis, restriction fragment length polymorphism, DHPLC, gene chip, and mass spectrometry). While each of these technologies has its advantages, they also tend to be time-consuming, tedious, costly, and overall not suitable for large-scale detection in clinical applications. EVA displays high genetic heterogeneity with a genetic diagnosis involving multiple known and unknown loci. Thus, good diagnoses require simultaneous high-throughput detection of multiple gene variants. Since its introduction in 2005, NGS has revolutionized genomic research by providing more cost-effective, faster, and more high-throughput sequencing than traditional technologies [49, 50]. Three main NGS platforms currently exist: Illumina/Solexa, Roche/454, and Life Technologies/SOLiD [51]. In this study, the target region capture system used multiple PCR enrichment with special reaction conditions in a PCR-based non-hybridization gene enrichment scheme. Multiple PCR enrichment technology can run 140–200 multiplex PCRs of 150–450 bp simultaneously. This system is easy to use and allows simple NGS library preparation. The capture range is small and amplified segments overlap to optimize cost and uniform coverage. This method is customizable for application in unique research cases. The NGS-based targeted sequencing method developed in this study could directly achieve nearly complete coverage of all coding regions of the three EVA genes. Furthermore, our results manifested that this sequencing technology is highly sensitive and specific in detecting sequence variants in these EVA genes. We propose that this NGS-based screening strategy is an effective alternative method to identify the multiple genetic causes of EVA that will improve the molecular diagnosis of EVA patients in clinical applications.
